# Mechanism of Astragalus Polysaccharide in Alleviating Bovine Mammary Fibrosis Through ROS/NLRP3 Inhibition and EMT Regulation

**DOI:** 10.3390/antiox14050503

**Published:** 2025-04-23

**Authors:** Jiang Zhang, Kejiang Liu, Tingji Yang, Hongwei Duan, Longfei Xiao, Quanwei Zhang, Yong Zhang, Weitao Dong, Xingxu Zhao

**Affiliations:** 1 College of Veterinary Medicine, Gansu Agricultural University, Lanzhou 730070, China; zj_m80@163.com (J.Z.); lkj1631996@163.com (K.L.); ytj19980227@163.com (T.Y.); grand6138@163.com (H.D.); zhychy@163.com (Y.Z.); 2 Gansu Provincial Key Laboratory of Animal Reproductive Physiology and Reproductive Regulation, Lanzhou 730070, China; zhangqw@gsau.edu.cn; 3 Veterinary Science (Traditional Chinese Medicine)—Municipal Laboratory of Beijing, Beijing University of Agriculture, Beijing 102206, China; xiaolf1989@bua.edu.cn; 4 College of Life Sciences and Technology, Gansu Agricultural University, Lanzhou 730070, China

**Keywords:** mastitis, fibrosis, astragalus polysaccharide, ROS/NLRP3, epithelial-mesenchymal transition

## Abstract

Mastitis in dairy cows, typically caused by bacterial infection, is a common inflammatory condition of the mammary tissue that leads to fibrosis, adversely affecting cow health, milk production, and dairy product quality. Astragalus polysaccharide (APS) has shown effectiveness in alleviating inflammation and fibrosis in various organs. The study employed lipopolysaccharide (LPS) to induce fibrotic conditions in two experimental systems: MAC-T bovine mammary epithelial cells and Kunming mouse models. Key parameters, including relative gene mRNA expression, protein levels, and reactive oxygen species (ROS) levels, were assessed using RT-qPCR, Western blotting (WB), and 2’,7’-Dichlorofluorescin diacetate (DCFH-DA) techniques, while histological analysis of mammary tissue was performed using H&E and Masson trichrome staining. Measuring malondialdehyde (MDA) levels, assessing the enzyme activities of catalase (CAT), and superoxide dismutase (SOD) were two methods of assessing oxidative stress. These methods were also tested in mouse mammary glands. APS significantly decreased ROS concentrations (*p* < 0.01), restored oxidative stress balance in mice (*p* < 0.05), and reduced fibrosis and inflammation, as demonstrated by histological observations and analysis. It also exerted regulatory effects on fibrosis markers (*E-cadherin*, *N-cadherin*, *α-SMA*) and inflammation markers (*NLRP3*, *ASC*, *Caspase-1*, *IL-1β*), as demonstrated by changes in their mRNA and protein expression. These findings endorse APS’s viability as an alternative therapeutic agent for mammary fibrosis therapy by demonstrating its ability to inhibit epithelial-mesenchymal transition (EMT) in vitro and mammary fibrosis in vivo, while also mitigating ROS production and reducing inflammation.

## 1. Introduction

Mastitis refers to the inflammation of the mammary glands of dairy cows, typically caused by bacterial infection. One of the most prevalent and serious illnesses in dairy farming, this condition not only compromises the health of the cows but also significantly lowers milk supply and quality [1]. Chronic mastitis closely linked to fibrosis in mammary tissues [2,3]. The epithelial-mesenchymal transition (EMT) is a significant factor in the development of mammary fibrosis, involving the transformation of epithelial cells into cells with mesenchymal traits [4]. This process results in the formation of fibroblast-like traits, excessive extracellular matrix (ECM) deposition, and morphological alterations. The morphology shifts from cobblestone to spindle, the epithelial cell polarity is lost, mesenchymal markers such as α-smooth muscle actin (α-SMA) and N-cadherin (CDH2) are upregulated, and the epithelial marker E-cadherin (CDH1)—which is essential for maintaining cell adhesion and lateral junctions—is downregulated. EMT is crucial for both pathological and physiological processes, with the conversion of epithelial cells into fibroblast-like cells being a defining characteristic of fibrosis.

In chronic inflammatory conditions, activated immune cells primarily generate reactive oxygen species (ROS), resulting in oxidative stress and tissue damage [5], which contribute to the fibrotic process via several pathways [6]. For instance, diseases such as pulmonary fibrosis [7], hepatic fibrosis [8], renal fibrosis [9], and cardiac fibrosis [10] are closely associated with excessive ROS production. ROS are signaling molecules that, when they reach high intracellular levels, can activate the NLRP3 inflammasome. This activation recruits apoptosis-associated speck-like protein containing CARD (ASC) and activates caspase-1, promoting the maturation and secretion of IL-1β and IL-18, which are essential mediators of the inflammatory response [11,12], These factors play a critical role in the initiation of both local and systemic inflammatory responses [13]. Inhibiting ROS production and NLRP3 activation has been shown to effectively attenuate inflammation-induced fibrosis [14].

The powerful tonic and immunomodulatory properties of Astragalus polysaccharide (APS), an active polysaccharide that is derived from Astragalus membranaceus, have been acknowledged in traditional Chinese medicine. APS possesses a broad spectrum of bioactivities, particularly in immune regulation, anti-inflammation, antioxidant, and anti-fibrotic effects. It is extensively utilized in the treatment of cardiovascular illnesses, diabetes, cancer, respiratory disorders, and neurological ailments [15,16,17,18]. APS has also demonstrated therapeutic potential in various LPS-induced chronic inflammation-related disorders [19], cellular inflammation models [20], and fibrotic diseases such as hepatic fibrosis [21], myocardial fibrosis [22], and renal fibrosis [23]. In therapeutic applications, APS promotes thymocyte proliferation, reduces drug-induced apoptosis, and improves immune function in immunosuppressed mice by increasing thymic index and immunoglobulin levels while inhibiting IL-2 overproduction [24]. APS inhibits pro-apoptotic proteins, including BAX, while promoting the overexpression of anti-apoptotic proteins such as BCL-2 and BCL-X2 in cancer models by blocking mitochondrial apoptosis pathways [25].

**Figure 1:**
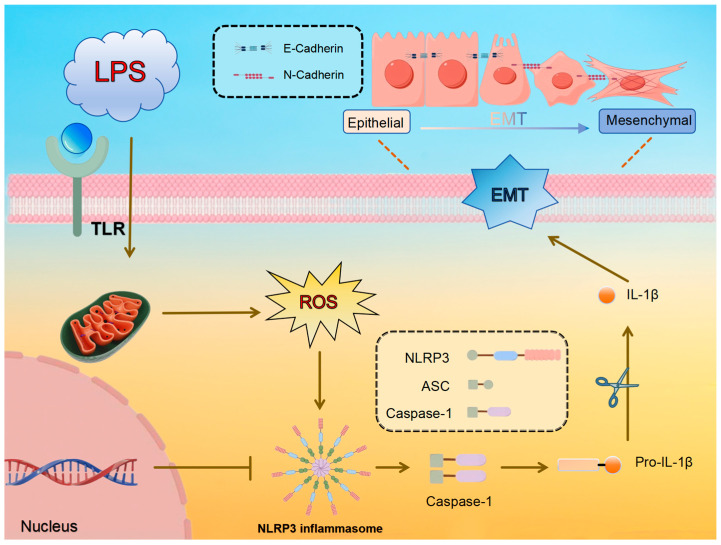
Predicted molecular mechanism of LPS-induced ROS/NLRP3 regulation of EMT in mammary epithelial cells.

This research aims to investigate the potential of APS in reducing LPS-induced mammary fibrosis by modifying NLRP3 activity and inhibiting ROS generation, as measured in both cellular and animal models. The study investigates the molecular processes behind its protective benefits mammary glands of dairy cows, offering information and resources for the creation of fresh treatment approaches and prophylactics against mammary fibrosis. The pathway diagram involved in this experiment is shown in Figure 1.

## 2. Materials and Methods

### 2.1. Cell Samples

The MAC-T cells were obtained from our research group and cultured according to the method described by Wang et al. [26]. The cells were subsequently subjected to various concentrations of LPS (12.5, 25, and 50 µg/mL) and APS (25, 50, and 100 µg) for a duration of 24 h. The source of the APS was Solarbio (Beijing, China), while the LPS was obtained from Sigma (Shanghai, China). In inhibition studies, MCC950 (an NLRP3 inhibitor, Macklin, Shanghai, China) was used at 10 µM and incubated for two hours before treatment, alongside 50 µg/mL LPS. Three biological replicates were used in the in vitro cell experiments.

### 2.2. Animal Samples

In this study, 90 eight-week-old female Kunming mice were used as the research subjects, and the feeding standards referred to the method of Fang et al. [27]. After seven days of breastfeeding, five groups of ten mice each were created from the selection of pregnant mice with similar body conditions: the control group, the LPS group (200 µg/mL, Solarbio, Beijing, China), and the APS group (50, 100, and 200 mg/kg). All of the remaining mice were divided into four groups: Control, APS, LPS, and APS+LPS. All of the mice received injections into the milk ducts of the fourth pair of mammary glands: 50 µL of saline was given to the control group, 50 µL of 200 µg/mL LPS was given to the APS group, and APS+LPS received both LPS injections and APS gavage at the appropriate doses. Gavage treatment commenced on the first day of lactation. On day six, all animals were administered intraperitoneal pentobarbital sodium (150 mg/kg) to induce whole-body anesthesia, followed by euthanasia. For analysis, one portion of the removed mammary glands was promptly frozen at −80 °C, while the other portion was preserved in 4% paraformaldehyde for histological investigation. The study was carried out in accordance with ethical guidelines that were authorized by Gansu Agricultural University’s Animal Protection Committee (GSAU-Eth-VMC-2021-020).

### 2.3. RT-qPCR

MAC-T cells and mouse mammary gland tissues were cultured, collected, and washed with PBS. Total RNA extraction from MAC-T cells and mouse mammary gland tissues was performed using TRIzol reagent (Transgen, Beijing, China). Subsequent chloroform shaking and centrifugation were performed, followed by isopropanol addition for RNA precipitation and washing. Finally, RNA concentration and purity were assessed. Quantitative PCR was employed to assess the expression levels of fibrosis indicators *E-cadherin (CDH1 gene)*, *N-cadherin (CDH2 gene)* and *α-SMA (ACTA2 gene)*, with inflammatory markers *NLRP3*, *ASC (PYCARD gene)*, *Caspase-1*, and *IL-1β*. Primers for *GAPDH* as an internal reference for qPCR were designed and synthesized using Primer Premier 3.0 (Qingke, Beijing, China) (Table 1). RT-qPCR was performed following the method described by Wang et al. [26].

### 2.4. Western Blot

Cold RIPA buffer was used to extract proteins from mammary tissues and cells samples (Solarbio, Beijing, China). The expression of fibrosis indicators and inflammation in mammary tissue was assessed by Western blot (WB) analysis. Primary antibodies, including E-cadherin (1:20,000), N-cadherin (1:2000), α-SMA (1:1000), and NLRP3 (1:1000) by Proteintech (Wuhan, China), ASC (1:1000) by ABclonal (Wuhan, China), and Caspase-1 (1:1000), and IL-1β (1:1000) by Abmart (Shanghai, China), against were incubated with the samples overnight at 4 °C. The samples were treated with the appropriate secondary antibodies (goat anti-rabbit IgG, 1:10,000; goat anti-mouse IgG, 1:10,000; SAB, Greenfield, MD, USA) for one hour at 37 °C. All protein bands were seen using an enhanced chemiluminescence (ECL, Solarbio, Beijing, China). ImageJ software (version 1.52a) was used to quantify and evaluate band intensities in order to quantify signals, with β-actin used as a normalization control.

### 2.5. Determination of Mitochondrial ROS Levels

To find the total cell ROS level, the Reactive Oxygen Assay Kit (Solarbio, Beijing, China) was utilized. MAC-T cells were first injected onto 6-well plates, and the necessary procedures were then followed. The cells were subsequently rinsed gently with PBS three times following a 30-min treatment with 10 µmol/L DCFH-DA reagent at 37 °C, protected from light. An inverted fluorescent microscope (Axiocam 208 colour, Zeiss, Oberkochen, Germany) was used to take the following pictures.

### 2.6. H&E and Masson Staining Analysis

Samples of mouse mammary tissue were preserved for 14 days in 4% paraformaldehyde. After fixation, samples were cleaned in xylene and paraffin-embedded, the tissues were washed under running water, and they underwent a series of graded alcohols to de-hydrate them. After being cut into 5 µm slices, paraffin blocks were stained using Masson’s trichrome and H&E. Using a Zeiss microscope and a slice scanner (Dynamax, Shanghai, China) to acquire images, dynamic histological alterations in the mouse mammary gland were detected.

**Table 1:** Primer information.

Gene Name	Species	Primer Sequence	Product Length	Tm/°C
*GAPDH*	*Cow*	F: GGAGCGAGACCCCACTAACAT	247	61
		R: TAAGGGGGCTAAGCAGTTGGT		
	*Mouse*	F: GGCTGTATTCCCCTCCATCG	154	60
		R: CCAGTTGGTAACAATGCCATGT		
*CDH1*	*Cow*	F: CAACAAGGAAACAGGCGTCA	175	59
		R: TGGGTTGAATCTGGGAGCAT		
	*Mouse*	F: GGCACTCTTCTCCTGGTCCTG	110	61
		R: AAGATGGTGATGATATGAGGCTGTG		
*CDH2*	*Cow*	F: CAGTGTGATTCCAACGGGGA	146	60
		R: TCCCGGCGTTTCATCCATAC		
	*Mouse*	F: ACAGCCCCTTCTCAATGTGA	231	59
		R: TCAGGTAGGGCTGGTTTGAG		
*ACTA2*	*Cow*	F: ACCATCGGGAATGAGCGTTT	97	60
		R: TGTTGTACGTGGTCTCGTGG		
	*Mouse*	F: GCATGCAGAAGGAGATCACG	157	59
		R: TCGTCGTACTCCTGTTTGCT		
*NLRP3*	*Cow*	F: CAACGGGGAAGAGAAGGCAT	297	60
		R: TTGAGGTTCACGCTCTCACC		
	*Mouse*	F: GGCCAAAGAGGAATCGGACA	483	60
		R: CTACGGCCGTCTACGTCTTC		
*PYCARD*	*Cow*	F: TGAGCAAGGGCCCTAGAAAC	137	60
		R: ATCCAGAACCCCATCCACGA		
	*Mouse*	F: GTGAGCTCCAAGCCATACGA	124	60
		R: TGACAGTGCAACTGCGAGAA		
*Caspase-1*	*Cow*	F: ACAGCTATGGATAGAGCCCGA	135	60
		R: ACTTTCTGAAGTGAGCCCCAG		
	*Mouse*	F: TCCTTGTTTCTCTCCACGGC	124	60
		R: CGAGGGTTGGAGCTCAAGTT		
*IL-1β*	*Cow*	F: TCCGACGAGTTTCTGTGTGA	206	59
		R: ATACCCAAGGCCACAGGAAT		
	*Mouse*	F: GGAGCCTGTAGTGCAGTTGT	208	60
		R: AGCTTCAGGCAGGCAGTATC		

### 2.7. Detection of Mammary Gland Oxidative Stress Levels

The Malondialdehyde (MDA) assay kit (Solarbio, Beijing, China) was utilized to assess lipid oxidation, while the Superoxide Dismutase (SOD) activity assay kit (Solarbio, Beijing, China) was employed to quantify the activity of the enzyme responsible for H_2_O_2_ production. The activity of H_2_O_2_-scavenging enzymes is assessed using the Catalase (CAT) activity test kit (Solarbio, Beijing, China).

### 2.8. Data Processing and Statistical Analysis

All data were analyzed using SPSS 22.0 (SPSS Inc. Chicago, IL, USA). Select mean ± SEM to represent values. Statistical significance was determined by one-way ANOVA, and Figures were prepared using GraphPad Prism 9.0 (GraphPad Software Inc., San Diego, CA, USA), with * and # indicating *p* < 0.05, and ** and ## representing *p* < 0.01. * compares to LPS, # to control.

## 3. Results

### 3.1. APS Suppresses LPS-Induced Epithelial-Mesenchymal Transition in MAC-T Cells

**Figure 2:**
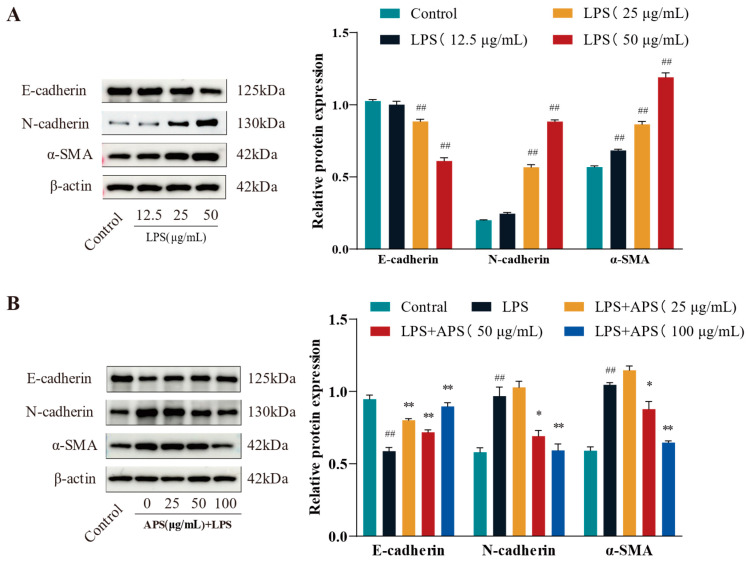
APS prevents LPS-induced EMT in MAC-T cells, according to WB analysis of N-cadherin, E-cadherin, and α-SMA. (**A**) LPS (12.5, 25, and 50 µg/mL) was administered to MAC-T cells for a duration of 24 h. (**B**) For 24 h, MAC-T cells were exposed to 50 µg/mL of LPS and varying concentrations of APS (25, 50, and 100 µg/mL). ^##^ *p* < 0.01 vs. the Control group, ** *p* < 0.01, * *p* < 0.05 vs. the LPS group.

To evaluate fibrosis marker expression and identify the optimal LPS dosage for inducing fibrosis, cells were exposed to lipopolysaccharide (LPS) at doses of 12.5, 25, and 50 μg/mL. As illustrated in Figure 2A, treatment with 50 µg/mL LPS led to a marked reduction in E-cadherin levels, alongside significant upregulation of α-SMA and N-cadherin (*p* < 0.01), relative to the control group. MAC-T cells were administered 50 µg/mL LPS alongside 0, 25, 50, and 100 µg/mL APS to evaluate its potential in mitigating LPS-induced fibrosis. The results were juxtaposed with the control group. The results in Figure 2B indicated that APS treatment exerted a concentration-dependent impact, characterized by an increase in E-cadherin expression and a decrease in N-cadherin and α-SMA expression. Notably, treatment with 100 µg/mL APS significantly upregulated E-cadherin while downregulating N-cadherin and α-SMA levels (*p* < 0.01), in contrast to the group under authority.

### 3.2. Inhibiting ROS Generation by APS Reduces LPS-Induced EMT in MAC-T Cells

**Figure 3:**
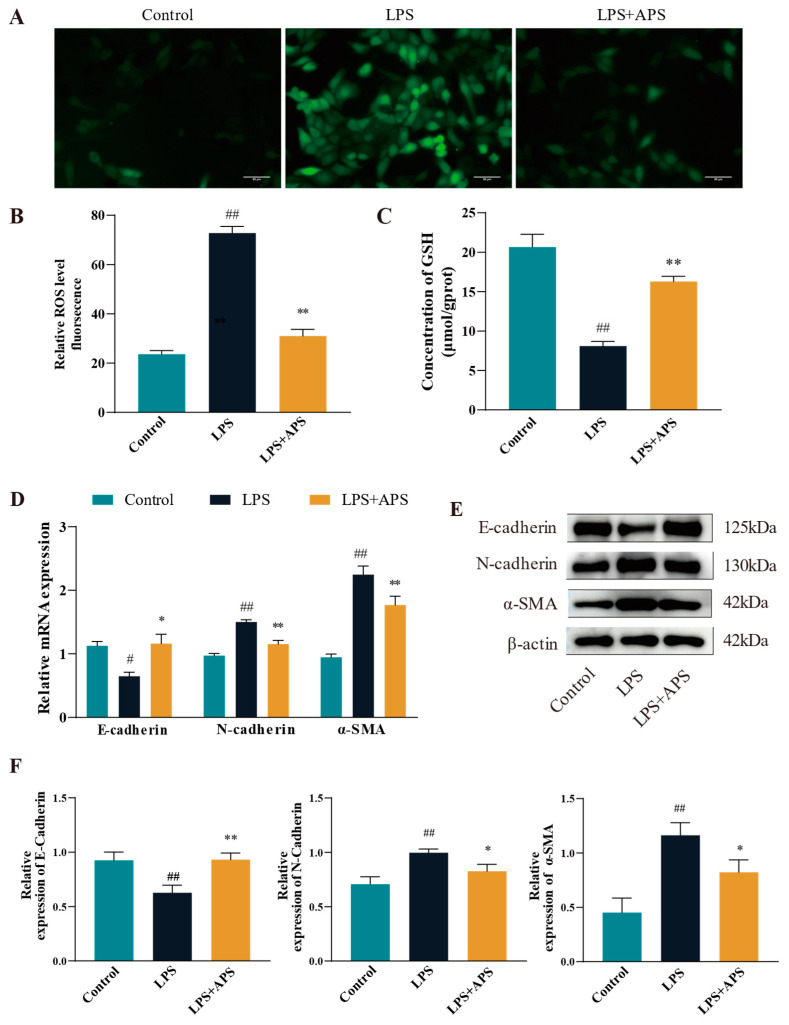
By inhibiting ROS in MAC-T cells, APS reduces LPS-induced EMT. (**A**) To treat mac-t cells, 50 µg/mL LPS and 100 µg/mL APS were used. Cells received a 24-h treatment. (**B**) DCFH-DA assay for total reactive oxygen species content (scale bar = 50 µM). (**C**) GSH levels. (**D**–**F**) qPCR was used to identify *E-cadherin*, *α-SMA*, and *N-cadherin* mRNAs and proteins linked to EMT with WB. ^##^ *p* < 0.01, ^#^ *p* < 0.05 vs. the Control group, ** *p* < 0.01, * *p* < 0.05 vs. the LPS group.

To evaluate the impact of APS on ROS levels in LPS-stimulated MAC-T cells, ROS fluorescence signals and glutathione (GSH) enzyme activities were measured. The ROS positivity was significantly reduced (*p* < 0.01) in the APS-treated group relative to the LPS-treated group, as seen in Figure 3A–C. It was found that the antioxidant enzyme content was reduced in the LPS-treated group by detecting GSH content (*p* < 0.01), while GSH significantly rebounded after co-incubation with APS (*p* < 0.01). The expression of fibrogenic markers at the mRNA and protein levels was analyzed using qPCR and Western blot to determine if APS mitigates LPS-induced fibrosis by inhibiting ROS production. As for gene expression compared with the LPS group, the mRNA levels of *N-cadherin* and *α-SMA* were significantly lower (*p* < 0.01), while *E-cadherin* expression was significantly increased (*p* < 0.05) in the APS-treated group (Figure 3D). Figure 3E,F Protein analysis indicated results consistent with mRNA, as the APS-treated group had a significant decrease (*p* < 0.05) in N-cadherin and a-SMA expression, with a notable rise (*p* < 0.01) in E-cadherin expression.

### 3.3. Effects of APS on the NLRP3 Pathway Mitigates the Process of Cellular EMT

**Figure 4:**
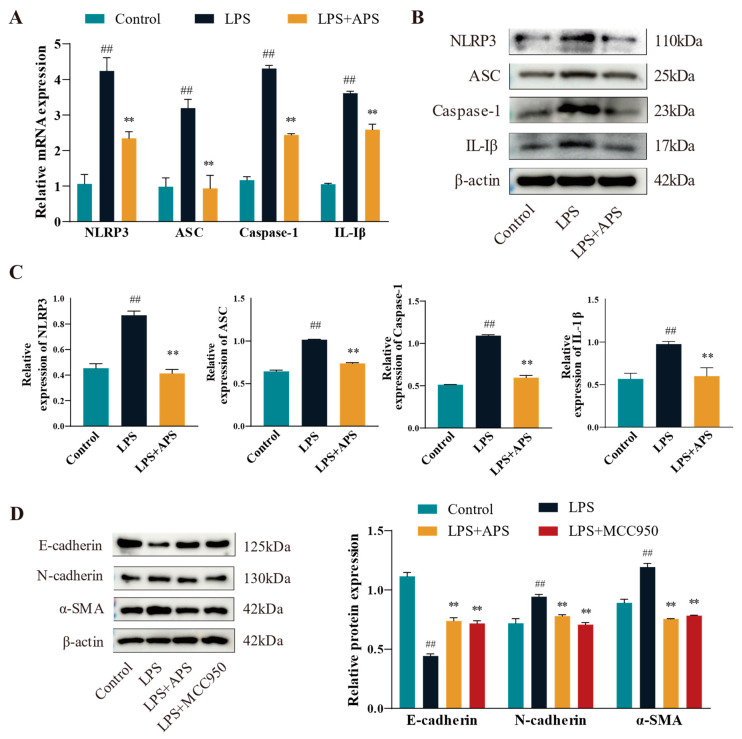
APS suppresses fibrotic factors and influences the NLRP3 inflammatory pathway. (**A**–**C**) *NLRP3*, *ASC*, *Caspase-1*, and *IL-1β* expressions were found by qPCR and WB. (**D**) After MAC-T cells were exposed to 100 μg/mL astragalus polysaccharide, 10 μM MCC950, and 50 μg/mL LPS for a full day, the WB assay E-cadherin, N-cadherin, and α-SMA expression levels were determined using this method. ^##^ *p* < 0.01 vs. the Control group, ** *p* < 0.01 vs. the LPS group.

This study assessed the changes in mRNA expression (Figure 4A) and protein levels (Figure 4B,C) of inflammatory factors (*NLRP3*, *ASC*, *Caspase-1*, and *IL-1β*) in various groups in order to determine whether APS affects the NLRP3 pathway (with decreased expression) and ultimately lessens the degree of cellular EMT. Compared to the LPS group, expression levels of inflammatory markers in the APS group were significantly reduced (*p* < 0.01). To further investigate whether NLRP3 plays an important role in cell fibrosis, cells were incubated with MCC950 as a specific inhibitor of NLRP3 and APS simultaneously to detect fibrosis marker proteins. WB bands demonstrated that, in comparison to the LPS group, the expression levels of E-cadherin, N-cadherin, and a-SMA proteins were considerably recovered in the MCC950 and APS groups (*p* < 0.01) (Figure 4D).

### 3.4. APS Inhibition of Mammary ROS and EMT Study in Mice

**Figure 5:**
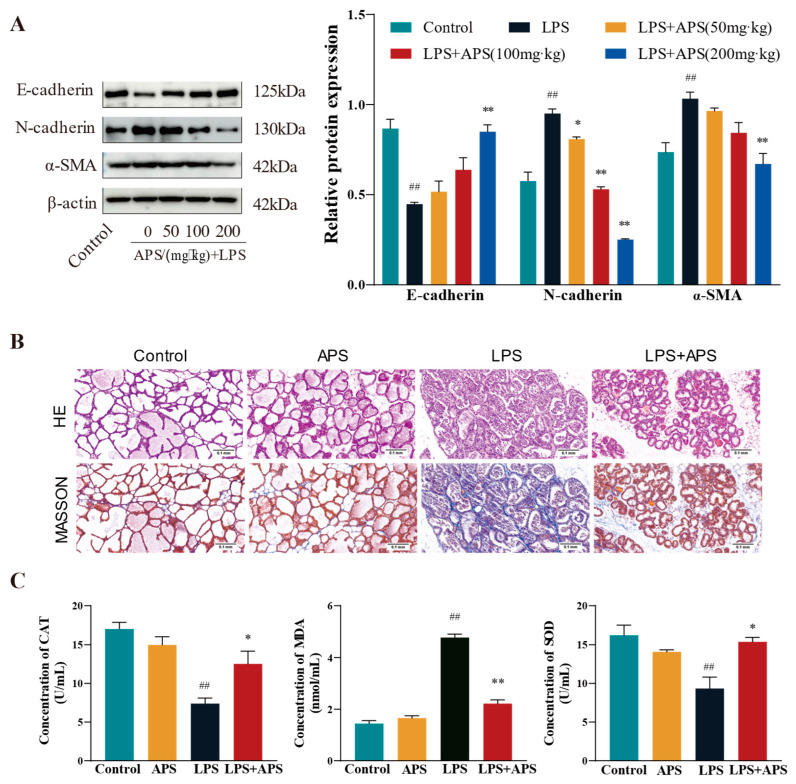
APS inhibits mammary ROS and EMT progression in mice. (**A**) Gavage with varying doses of APS (50, 100, and 200 mg/kg) following LPS treatment (200 µg/mL). Western blot analysis to quantify the expression levels of fibrosis-related proteins α-SMA, N-cadherin, and E-cadherin. The enrolled mice were split into four groups: Control, APS, LPS, and LPS+APS groups. (**B**) Mammary tissue stained by H&E and Masson. (**C**) CAT, MDA, and SOD contents in mammary tissues. ^##^ *p* < 0.01 vs. the Control group, ** *p* < 0.01, * *p* < 0.05 vs. the LPS group.

In this study, we investigated how varying APS concentrations affected the expression of associated variables in the fibrosis model. E-cadherin protein expression was substantially higher (*p* < 0.01) in the APS group than in the LPS group, but N-cadherin and α-SMA expression was significantly lower (*p* < 0.01) in the APS group (Figure 5A). Both mammary gland structure of the control and APS groups was found to be normal based on the findings of H&E and Masson staining (Figure 5B). In contrast, the LPS group exhibited an incomplete basal layer structure of the mammary gland with increased neutrophilic and fibrous components. APS treatment provided a better protective effect against LPS-induced mastitis. Masson staining also revealed aberrant collagen fibre deposition in mouse mammary tissue, and following APS therapy, there was a substantial decrease in blue fibrous tissue. Regarding oxidative stress markers (Figure 5C), there were no appreciable variations in the levels of MDA, SOD, and CAT in the mammary glands between the control and APS groups. In comparison to the control group, LPS significantly increased the levels of MDA lipid oxidation in mammary tissues and significantly decreased the activity of the CAT and SOD enzymes, which were markers of oxidative stress (*p* < 0.01). MDA levels considerably decreased (*p* < 0.01) during APS treatment, but CAT and SOD levels markedly rose (*p* < 0.05).

### 3.5. APS Attenuated Mouse Mammary Fibrosis Triggered by NLRP3 Activation

**Figure 6:**
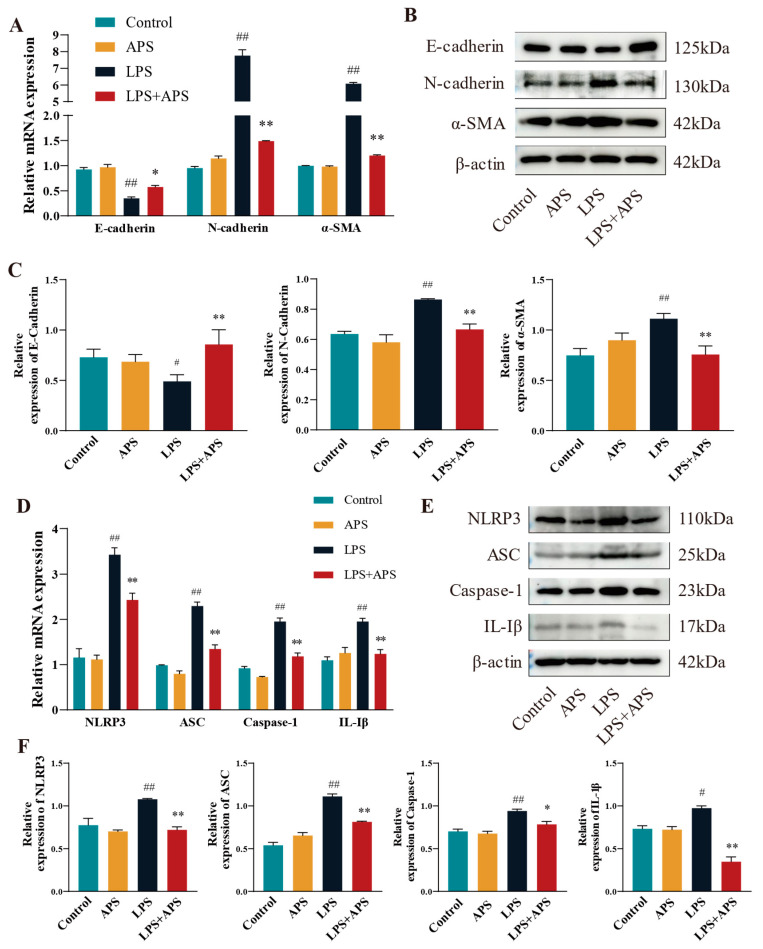
In mouse mammary tissue APS inhibited fibrosis by affecting NLRP3 activation and thereby inhibiting fibrosis. Using qPCR (**A**–**D**) and WB (**B**,**C**,**E**,**F**), the mRNA and protein expression of EMT factors (*E-cadherin*, *N-cadherin*, and *α-SMA*) and inflammatory factors (*NLRP3*, *ASC*, *Caspase-1*, and *IL-1β*) were identified. ^##^ *p* < 0.01, ^#^ *p* < 0.05 vs. the Control group, ** *p* < 0.01, * *p* < 0.05 vs. the LPS group.

When NLRP3 was activated in mice, APS reduced the amount of LPS-induced mammary fibrosis. This study examined the mRNA and protein expression of fibrotic and inflammatory elements in mouse mammary tissue utilizing RT-qPCR and Western blot techniques. The results demonstrated no significant differences between the control and APS groups. A significant difference (*p* < 0.01) in mRNA expression was noted between the LPS group and the Control group. Fibrosis factors (*N-cadherin*, *α-SMA*) and inflammatory factors (*NLRP3*, *ASC*, *Caspase-1*, and *IL-1β*) were significantly reduced in the mammary tissue of LPS+APS group, while the expression of the epithelial marker *E-cadherin* increased markedly (Figure 6A–D). E-cadherin was dramatically diminished (*p* < 0.05) and IL-1β was markedly elevated (*p* < 0.05) in the LPS group compared to the Control group, as per WB analysis of protein expression (Figure 6B,C,E,F). Other components exhibited a statistically significant increase (*p* < 0.01), including N-cadherin, α-SMA, NLRP3, ASC, and Caspase-1. Conversely, the residual inflammatory and fibrotic elements were markedly restored (*p* < 0.01), and Caspase-1 expression was dramatically reduced in the APS treatment group compared to the LPS group (*p* < 0.05).

## 4. Discussion

Mastitis in dairy cows presents a significant threat not only to the health of the cows but also to the quality of dairy products, economic efficiency, and reproductive capacity [28]. Inflammation and fibrosis are closely linked physiological processes. Studies have shown that chronic inflammation, especially due to prolonged activation of the immune system, triggers excessive deposition of cellular matrices such as collagen while promoting excessive proliferation of fibroblasts, ultimately contributing to fibrosis development [2,29]. As a calcium-dependent transmembrane protein, the core functions of E-cadherin are manifested in three aspects: mediating intercellular adhesion, maintaining the epithelial polarity structure, and ensuring the structural integrity of mammary tissue [30]. The reduction in E-cadherin results in diminished cell polarity and intercellular adhesion during the EMT, signifying a loss of epithelial traits. The overexpression of N-cadherin enhances cell migration and infiltration, but elevated α-SMA expression often indicates the attainment of mesenchymal characteristics, rendering the cells contractile and engaged in tissue remodeling and fibrosis [4]. The concurrent expression of these proteins in epithelial cells frequently indicates heightened migratory proficiency and an augmented capability for extracellular matrix secretion. The major pro-inflammatory component of Gram-negative bacteria, LPS is a strong innate immune stimulator that can induce tissue fibrosis [31,32]. In particular, LPS was utilized in the construction of a cow endometrial epithelial cell line (BEND) [33] and a mouse adipose tissue fibrosis model [34]. Thus, LPS was utilized as a virulence factor to establish a fibrosis model in this study. The results demonstrated that E-cadherin expression dropped as LPS concentration rose, whereas N-cadherin expression increased with α-SMA. This suggested that LPS would cause fibrosis in MAC-T cells. Numerous clinical and experimental studies have validated APS’s anti-inflammatory and antifibrotic properties [13], especially the ability to inhibit stellate cell LX-2 fibrosis in the liver [35] and NRK-52E, a rat renal tubular epithelial cell [36]. This study involved the induction of MAC-T cells in vitro with LPS to establish a fibrosis model. APS treatment increased the expression of the epithelial cell marker E-cadherin and decreased the levels of the mesenchymal markers N-cadherin and a-SMA. As both an antioxidant and anti-inflammatory agent, APS not only inhibited LPS-induced ROS production in osteoblasts [37], but also protected chick embryo fibroblasts from autophagic damage by reducing ROS levels [38]. ROS, known for their strong oxidizing properties, are pivotal in the onset and advancement of various diseases. The buildup of ROS beyond normal levels leads to oxidative stress, a key mechanism contributing to chronic inflammation [5]. The buildup of ROS is not only a hallmark of inflammation but also a key factor in tissue and organ fibrosis [6]. The development of several illnesses has been closely linked to the excessive buildup of ROS [39]. It has been shown that the fibrotic process in the liver is closely affected by the excessive accumulation of ROS, with accelerated activation and over-proliferation of hepatic stellate cells, and enhanced capacity to secrete extracellular matrix, which ultimately results in the manifestation of fibrotic symptoms in the liver [40]. Consequently, the timely removal of accumulated ROS can help mitigate fibrosis [41].

Moreover, it has been demonstrated that ROS levels are markedly increased when cells experience oxidative stress. The increase in ROS activates and interacts with the NLRP3 receptor either directly or through oxidative modification of other molecules to promote NLRP3 polymerisation to co-assemble with ASC proteins and Caspase-1 to form inflammatory vesicles. The activation and recruitment of caspase-1 by inflammatory vesicles induce the maturation and release of the pro-inflammatory IL-lβ and IL-18 proteins. This induces an inflammatory response and alters the activation or activity of transcription factors such as Snail [42], Slug [43], and Twist [25]. These factors promote EMT in a variety of diseases, for example, they promote EMT in cancer stem cells (CSC) [44] and inhibit tumour growth. This study utilized MCC950 [45] as a potent and selective NLRP3 inhibitor. The results showed that APS had the same function as the NLRP3 inhibitor (MCC950), and the LPS-induced cell model decreased both α-SMA and N-cadherin protein expression and promoted E-cadherin expression. These findings align with results observed with MCC950 inhibitors in liver fibrosis [46], myocardial fibrosis [47], and renal fibrosis [48]. This supports the notion that APS’s antioxidant properties diminish tissue and organ fibrosis by inhibiting the activation of the NLRP3 inflammasome.

In periparturient cows, APS has demonstrated the ability to inhibit inflammation and reduce serum concentrations of IL-2, IL-6, and C-reactive proteins [49]. For early weaning yaks, Astragalus membranaceus extract can improve their antioxidant capacity and immunity [50]. The inclusion of Astragalus membranaceus in the diet of cashmere goats influences the levels of MDA, SOD, and CAT, therefore significantly reducing oxidation [51]. Supplementation of weaned lambs’ diet with APS significantly enhances their antioxidant capacity [52]. One of the major causes of fibrosis is the excessive buildup of ROS and chronic inflammatory stimuli, and the aforementioned data offer significant theoretical support for the role of APS in enhancing the antioxidant and anti-inflammatory capacity of ruminants. To determine whether APS operates via a similar mechanism in an LPS-induced mammary fibrosis animal model, we conducted validation experiments in mice. Consistent with data from diabetic mice with renal fibrosis, the results demonstrated that APS therapy repaired the structure of the mammary glands whereas LPS caused mammary fibrosis [36]. This implies that mouse mammary fibrosis can be treated with APS. In the LPS-induced mouse mammary model, APS reversed the mammary EMT process by decreasing the expression of N-adhesins and α-SMA and increasing the expression of E-adhesins. Recent studies have found that the development of organ fibrosis is closely influenced by inflammation and EMT. In peritonitis muscularis atrophic mice, APS significantly mitigated EMT in HMrSV5 cells and peritoneal fibrosis [53]. In this study, the expression of inflammatory factors (NLRP3, ASC, Caspase-1, and IL-1β) in LPS group was considerably higher than that of the Control group. Following APS therapy, all of these expressions exhibited a declining trend. These outcomes support the findings in rats with allergic rhinitis, further proving that APS inhibits the activation of NLRP3 inflammatory vesicles in rats, hence lowering the production of inflammatory markers (Caspase-1, IL-1β, and ASC). Another study demonstrated that APS effectively improved nasal mucosal inflammation in rats by down-regulating NLRP3 [54]. Thus, our findings imply that APS has a similar positive impact in animal models.

## 5. Conclusions

In this study, we showed that APS had a therapeutic impact on mammary fibrosis of mice and MAC-T cells that were generated by LPS. It ultimately reduced the EMT process primarily by preventing the overproduction of ROS and controlling the ROS/NLRP3 signaling pathway with the expression of inflammatory factors. Therefore, it was shown that APS has therapeutic potential for treating mammary fibrosis in dairy cows.

## Data Availability

By writing the author and corresponding author, you may request access to all of the raw data produced during this study.

## Abbreviations

The following abbreviations are used in this manuscript:

APS	Astragalus polysaccharide
CAT	Catalase
EMT	Epithelial-Mesenchymal Transition
GSH	Glutathione
LPS	Lipopolysaccharide
MAC-T	Bovine Mammary Epithelial Cell Line
MDA	Malondialdehyd
ROS	Reactive Oxygen Species
SOD	Superoxide Dismutase
